# The impact of repeated drug desensitisation on quality of life in drug hypersensitivity

**DOI:** 10.1002/clt2.70029

**Published:** 2025-02-17

**Authors:** Begum Gorgulu Akin, Secil Kepil Ozdemir, Beyza Doganay Erdogan, Asli Gelincik, Ozlem Goksel, Adile Berna Dursun, Sacide Rana Isık, Semra Demir, Hatice Serpil Akten, Sevim Bavbek

**Affiliations:** ^1^ Department of Chest Diseases Division of Allergy and Immunology Ankara University School of Medicine Ankara Turkey; ^2^ Immunology and Allergy Ankara Bilkent City Hospital Ankara Turkey; ^3^ Department of Chest Diseases Division of Allergy and Immunology Dr. Suat Seren Chest Diseases and Surgery Training and Research Hospital University of Health Sciences Izmir Turkey; ^4^ Department of Chest Diseases Izmir Faculty of Medicine University of Health Sciences Izmir Turkey; ^5^ Department of Biostatistics Ankara University School of Medicine Ankara Turkey; ^6^ Istanbul Faculty of Medicine, Immunology and Allergic Diseases Istanbul University Istanbul Turkey; ^7^ Department of Chest Diseases Division of Allergy and Immunology Ege University School of Medicine Izmir Turkey; ^8^ School of Medicine, Immunology and Allergic Diseases Lokman Hekim University Ankara Turkey; ^9^ Koc Healthcare American Hospital Istanbul Turkey

**Keywords:** allergy, chemotherapy, desensitisation, drug, drug hypersensitivity, Drug Hypersensitivity Quality of Life Questionnaire, quality of life

## Abstract

**Background:**

Measurement of disease‐specific quality of life (QOL) is crucial in evaluating the effects of disease and response to treatment. Patients' efforts to avoid the responsible medication can have a negative impact on the QOL of patients with drug hypersensitivity reactions (DHRs). The Drug Hypersensitivity QOL Questionnaire (DrHy‐Q) is the only specific tool measuring disease specific QOL in patients with DHRs.

**Objective:**

To evaluate the effect of repeated drug desensitisation on disease‐specific QOL using the Turkish version of DrHy‐Q in a prospective multicentre study.

**Methods:**

Patients scheduled to undergo repeated desensitisations with the same drug were included in the study. Baseline DrHy‐Q scores were recorded for each patient prior to the commencement of the desensitisation procedure. DrHy‐Q scores were then calculated following each desensitisation procedure.

**Results:**

The study included 111 patients with two or more desensitisations (age mean ± SD, years: 53.87 ± 11.36, F/M:94/17). The drugs most implicated in DHRs were chemotherapeutics (91 of 111 patients, 82%) followed by biologicals (16 of 111 patients, 14.4%). Before the desensitisation process, the median (min–max) pre‐DrHy‐Q score was 39 (16–74). The median (min–max) DrHy‐Q scores after the first three desensitisation were 35 (19–69), 34 (15–68) and 35 (15–64), respectively. There was a statistically significant improvement in DrHy‐Q scores after the first three desensitisation in comparison with baseline.

**Conclusion:**

The health‐related disease‐specific QOL of patients with hypersensitivity to drugs significantly improved after the first three, but not after subsequent drug desensitisations, as compared to baseline.

## 1 INTRODUCTION

Drug hypersensitivity is one of the most common allergic diseases. The prevalence of self‐reported drug hypersensitivity in the community varies between 7% and 20%, depending on the average age of the groups studied and whether they are inpatient or outpatient.^1^, ^2^ In most patients with drug hypersensitivity reactions (DHRs), the symptoms are caused by exposure to the responsible drug and the reaction usually disappears after a period when the medication is discontinued. However, the absence of persistent symptoms does not necessarily indicate that the patient's quality of life (QOL) is not affected. Patients' efforts to avoid the responsible medications, anxiety and fear of the recurrence of the allergic reaction and concerns about using other drugs can have negative impacts on health related QOL. In chronic diseases, fearing that the disease will not heal or be adequately treated due to the inability to use drugs may also importantly impact QOL. Furthermore, DHRs may have psychological impacts.^3^

Measurement of disease‐specific QOL is important in evaluating the effects of diseases and response to treatments. QOL questionnaires are used as an endpoint in routine clinical practice and clinical research in allergic diseases as well as in other chronic diseases. Health‐related QOL questionnaires can be general or disease‐specific.^4^, ^5^, ^6^ As DHRs result in intermittent symptoms as a consequence of exposure to the culprit drug, it is not sufficient to assess the effects of DHRs on QOL using general QOL questionnaires. The only specific tool measuring health‐related QOL in patients with DHRs was developed by an Italian group in 2011 (Drug Hypersensitivity Quality of Life Questionnaire [DrHy‐Q]).^7^ The Turkish version of DrHy‐Q was found reliable and valid for evaluating QOL in patients with DHRs in 2016 (Table S1).^4^ In addition to the 15‐item standard DrHy‐Q, currently a 6‐item shorter version (DrHy‐Q‐6) was developed by Mak et al. in 2024.^8^

Drug desensitisation is defined as the establishment of a temporary state of tolerance to the drug responsible for the DHR and is achieved by administering gradually increasing doses of the relevant drug over a short period of time (hours to a few days) until the total cumulative therapeutic dose is reached. It is a high‐risk procedure and is only used in patients for whom alternative treatments are not available or, even if available, are less effective, taking into account the risk/benefit ratio.^9^

DrHy‐Q has been shown to be responsive to diagnostic interventions (skin tests and/or provocation tests), and provocation tests for finding safe alternative drugs.^3^, ^10^ Moreover, for the first time, we previously showed that disease‐specific QOL of patients with drug hypersensitivity significantly improves after drug desensitisation.^11^ This improvement was not affected by the implicated drugs, indications of the implicated drugs, severity of the initial DHR, and development of a breakthrough reaction (BTR) during desensitisation. The temporary tolerance induced by drug desensitisation is maintained by continuous administration of the medication. For treatments like chemotherapy or biologicals which are applied at specific intervals, desensitisation procedure must be repeated for every course. Thus, drug desensitisation in some cases is a repetitive process; however, there are no data on the effects of repeated drug desensitisation on disease specific QOL in patients with multiple drug desensitisation. Therefore, we aimed to evaluate the effect of repeated drug desensitisation on disease‐specific QOL using the Turkish version of DrHy‐Q in this prospective multicentre study.

## 2 MATERIALS AND METHODS

### 2.1 Drug Hypersensitivity Quality of Life Questionnaire (DrHy‐Q)

DrHy‐Q includes 15 items evaluated on a five‐point Likert scale (from 1 [not at all] to 5 [very much]). It was designed to be completed by the patient, is easy to administer and score and requires a few minutes to complete. Questions and scores were formulated so that higher scores reflected worse disease‐specific QOL.^7^ The validated Turkish version of the DrHy‐Q was used for evaluating the effect of drug desensitisation on disease‐specific QOL in this study.^4^

### 2.2 Drug desensitisation

Drug desensitisation indications and contraindications were determined in accordance with the international guidelines.^9^ Individual risk‐benefit evaluation was performed in every patient, and drug desensitisation was applied only when the benefits were thought to outweigh the risks. Desensitisation procedures were performed in adequately controlled settings in the allergy centres experienced in drug desensitisation under the supervision of a well‐trained allergist. While determining desensitisation protocols, available published protocols were taken into account whenever possible.^9^, ^12^, ^13^ All drug desensitisation protocols were performed in inpatient settings using the suggestion of Brigham and Women's Hospital (BWH)'s rapid drug desensitisation (RDD) protocol. The desensitisation protocol used was the 3‐bag/12‐step desensitisation protocol developed by BWH.^14^ Protocols for acetylsalicylic acid desensitisation are based on the position paper by Kowalski et al.^15^

### 2.3 Study design

The study, coordinated by the Ankara University Department of Allergy and Immunology, was conducted in the allergy and immunology clinics of 6 centres in Turkey, in accordance with the tenets of the Declaration of Helsinki after receiving approval from the local ethics committee (Approval date and number: 04/03/2021 and I2‐167‐21). The inclusion criteria were an age of at least 18 years, to have objective symptoms compatible with DHR, a diagnosis of DHR made by an allergist experienced in drug hypersensitivity, and to have an indication of repeated drug desensitisation with the culprit drug. The exclusion criteria were not to have an indication for drug desensitisation and/or to have a contraindication to drug desensitisation (uncontrolled asthma, haemodynamic instability, uncontrolled cardiovascular disease, life threatening immuno‐cytotoxic reactions, vasculitis, bullous skin reactions like Stevens Johnson Syndrome or toxic epidermal necrolysis and drug reaction with eosinophilia and systemic symptoms). Written informed consent was obtained from all participants. DrHy‐Q was applied to the patients immediately before the first desensitisation and immediately after each desensitisation.

### 2.4 Statistical analysis

Descriptive statistics for the categorical data are presented as counts and percentages, and quantitative data are presented either as mean ± standard deviations or median and minimum‐maximum depending on assumptions of normality based on visual (histograms and probability graphs) and analytical methods (Shapiro‐Wilk tests). The Chi‐square or Fisher's exact test was used to compare categorical variables, as appropriate. Percentage change values for DrHy‐Q scores before and after desensitisation were calculated using the following formula: ([after first DrHy‐Q‐ before DrHy‐Q]/before DrHy‐Q) × 100.

A Wilcoxon test was employed to compare the repeated QOL scores of those who underwent two or more repeated desensitisation procedures. Scores of those who underwent one‐time desensitisation and those who underwent repeated desensitisation were compared using the Mann Whitney *U* test. Bonferroni corrected *p* values were given. *p* < 0.05 was considered statistically significant. Data analysis was performed using the SPSS 11.5 for Windows software package (SPSS Inc.).

## 3 RESULTS

### 3.1 Characteristics of the study population

A total of 268 (F/M: 219/49) patients with a mean age of 53.08 ± 12.49 years were evaluated with DrHy‐Q before and after drug desensitisation. The number of patients with two or more desensitisations was 111 (F/M: 94/17). The demographic and clinical characteristics of the patients who underwent single and two or more desensitisations are shown in Table 1. The most implicated drug groups to DHRs with a need of ≥2 drug desensitisations were chemotherapeutics (91 of 111 patients, 82.0%), biologicals (16 of 111 patients, 14.4%) and iron (4 of 111 patients, 3.6%). The most frequently desensitised drugs were carboplatin (*n*: 46), oxaliplatin (*n*: 27), docetaxel (*n*: 8), and rituximab (*n*: 6). BTRs during desensitisations were observed in 34.2% (*n*: 38) of the patients. They were often grade 1 (47.4%; *n*: 18), grade 2 (28.9%; *n*: 11), and grade 3 (23.7%; *n*: 9). We continued RDD in five of these nine patients despite grade‐3 BTR since there were no other effective alternative chemotherapy options for them.

**TABLE 1:** Demographic and clinical characteristics of the patients who underwent single desensitisation and two or more desensitisations.

		All desensitisations (*n*: 268)	Single desensitisation (*n*: 157)	≥2 desensitisations (*n*: 111)	*p* value
Age (years) (mean ± SD)		53.08 ± 12.49	52.52 ± 13.24	53.87 ± 11.36	0.38
Sex (*n* [%])	Female	219 (81.7)	125 (79.6)	94 (84.7)	0.18
	Male	49 (18.3)	32 (20.4)	17(15.3)	
Education (*n* [%])	Primary school	106 (39.6)	58 (36.8)	48 (43.2)	0.61
	Secondary school	33 (12.3)	20 (12.7)	13 (11.7)	
	High school	70 (26.1)	42 (26.8)	28 (25.2)	
	University	59 (22)	37 (23.6)	22 (19.8)	
Desensitised drugs (*n* [%])	Chemotherapeutic	202 (75.4)	111 (70.7)	91 (82.0)	**0.02**
	Biological	41 (15.3)	25 (16.0)	16 (14.4)	
	Others (NSAID, iron etc)	25 (9.3)	21 (13.3)	4 (3.6)	
Type of symptoms of the DHR (*n* [%])	Urticaria and/or AE	47 (17.5)	24 (15.3)	23 (20.7)	0.31
	Respiratory reaction	41 (15.3)	28 (17.8)	13 (11.7)	
	Anaphylaxis	137 (51.2)	66 (42.0)	71 (64.0)	
	Urticaria and URS	43 (16)	39 (24.9)	4 (3.6)	
Initial DHR grade (*n* [%])	I	24 (9)	15 (9.6)	9 (8.1)	0.36
	II	143 (53.4)	92 (58.6)	51 (45.9)	
	III	101 (37.7)	50 (31.8)	51 (45.9)	
Indication of the culprit drug (*n* [%])	Malignant disease	229 (85.4)	130 (82.8)	99 (89.2)	0.10
	Rheumatological/autoimmune disease	23 (8.6)	14 (8.9)	9 (8.1)	
	Others	16 (6)	13 (8.3)	3 (2.7)	
BTR during desensitisation (*n* [%])	Yes	78 (29.1)	40 (25.5)	38 (34.2)	0.07
BTR grade (*n* [%])	I	37 (47.4)	19 (47.5)	18 (47.4)	0.36
	II	27 (34.6)	16 (40)	11 (28.9)	
	III	14 (18)	5 (12.5)	9 (23.7)	
Psychiatric comorbidity (*n* [%])	Yes	33 (12.3)	18 (11.5)	15 (13.5)	0.70
Pre‐DrHy‐Q scale score (median [min; max])		39 (15–74)	39 (15; 73)	39 (16; 74)	0.98
Post‐1st desensitisation DrHy‐Q scale score (median [min; max])		34 (25–71)	34 (15; 71)	35 (19; 69)	0.98
Percentage change in DrHy‐Q after first desensitisation (%) (median [min; max])		−10^a^ (−68; 62)	−6.66^a^ (−68; 50)	−7.5^a^ (−66; 61)	0.45

*Note*: Bolded *p* < 0.05 was considered statistically significant.

Abbreviations: AE, angioedema; BTR, breakthrough reaction; DHR, drug hypersensitivity reaction; Pre‐DrHy‐Q scale score, DrHy‐Q score before starting desensitisation; URS, mild upper respiratory symptoms.

^a^ Negative values indicate a decrease in percentage, while positive values indicate an increase in percentage.

### 3.2 Change in DrHy‐Q scores of the patients after desensitisations

**FIGURE 1:**
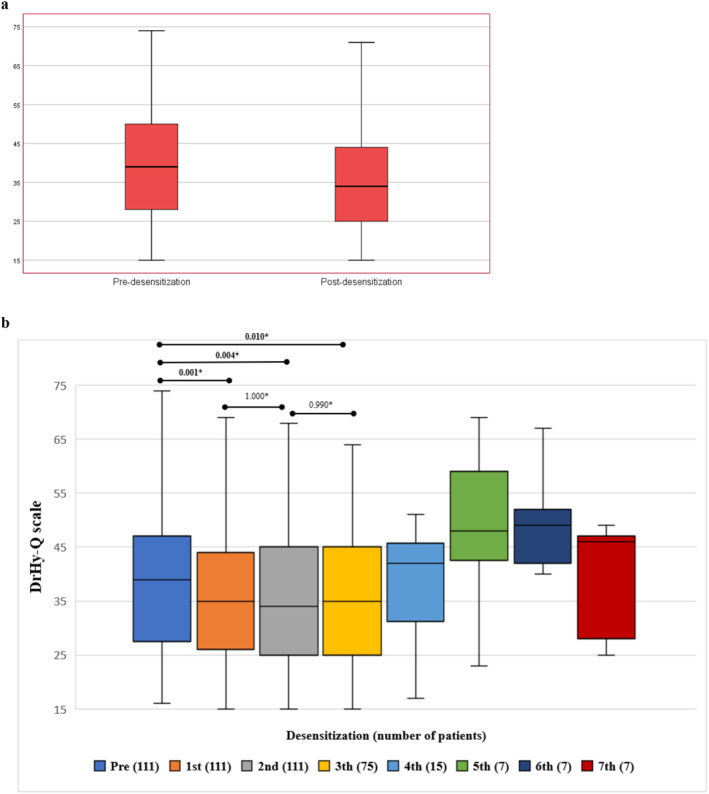
(A) Predesensitisation and postdesensitisation DrHy‐Q scale scores in 268 patients. The central box represents the values from the lower to the upper quartile (25th and 75th percentile), the line within the box indicates the median, and the whiskers show the minimum and maximum. (B) Change of DrHy‐Q scale scores in 111 patients with 2 or more desensitisations. *Bonferroni corrected *p* values. The central box represents the values from the lower to the upper quartile (25th and 75th percentile), the line within the box indicates the median, and the whiskers show the minimum and maximum. DrHy‐Q, Drug Hypersensitivity QOL Questionnaire.

Before starting the desensitisation process, the median Pre‐ DrHy‐Q scale score of 268 patients was (median [min–max]) 39 (15–74). The median (min–max) DrHy‐Q score after first desensitisation was 34 (15–71). There was a statistically significant improvement in QOL after the first desensitisation in comparison with baseline (*p*: 0.001) (Figure 1A). In 157 patients with only one desensitisation, the median percentage change in DrHy‐Q scale score was −6.6% (min; max −68%; 50%).

Pre‐ DrHy‐Q scale score of the 111 patients with two or more desensitisation was (median [min–max]) 39 (16–74). The median (min–max) DrHy‐Q score after first desensitisation was 35 (19–69). Percentage change in DrHy‐Q scale score after first desensitisation was median −7.5% (min; max −66%; 61%). After second desensitisation, the median (min–max) DrHy‐Q score was 34 (15–68). There was a statistically significant improvement in patients' QOL after the first three desensitisation in comparison with baseline (*p*: 0.001, 0.004, and 0.01, respectively). The post desensitisation DrHy‐Q scores after the fourth, fifth and sixth desensitisations were not statistically different from the baseline DrHy‐Q scores (Figure 1B).

A comparison of the DrHy‐Q scores after desensitisation between male and female patients revealed no statistically significant difference for the initial three desensitisations (*p* = 0.725, *p* = 0.217, *p* = 0.850), respectively (Table 2). Since there were no male patients in the fourth and subsequent desensitisations, no statistical analysis was performed. In addition, post‐desensitisation DrHy‐Q scores were compared according to the type of chemotherapy (platin vs. non platin) in 111 patients who underwent repeated desensitisations. No statistically significant difference was found (Table 2). We evaluated the effects of BTRs, psychiatric comorbidities and implicated drugs on post‐desensitisation DrHy‐Q scores (Table 2). Post‐desensitisation DrHy‐Q scores were significantly higher after the first, second, third and sixth desensitisations in patients with BTRs during desensitisations (*p*: 0.027, 0.015, 0.030 and 0.035, respectively). However, post‐desensitisation DrHy‐Q scores did not show any significant differences according to the grades of BTRs, existence of a psychiatric comorbidity, or type of the implicated drug (Table 2). All patients who underwent more than 3 desensitisation procedures were female; therefore, gender was not included in this assessment.

**TABLE 2:** Assessment of the possible factors affecting quality of life (QOL) after repeated drug desensitisations.

		DrHy‐Q scale score after desensitisation (median [min–max]), *p* value													
		1th		2nd		3th		4th		5th		6th		7th	
Sex	Female	35 (15–69)	0.725	35 (15–68)	0.217	35 (15–64)	0.850	44 (17–66)	‐	41 (17–69)	‐	48 (23–69)	‐	49 (26–67)	‐
	Male	32 (15–60)		30 (15–56)		35 (15–49)		32 (–)^a^		‐		‐		‐	
BTR during desensitisation	No	33 (15–63)	0.027*	32 (15–58)	0.015*	33 (15–64)	0.030*	44 (20–48)	0.690	37 (17–43)	0.220	43 (23–48)	0.035*	45 (26–50)	0.170
	Yes	37 (16–69)		36 (15–68)		41 (15–61)		47 (17–66)		51 (29–69)		67 (51–69)		54 (44–67)	
Grade of BTR	Grade 1	35 (15–69)	0.690	34 (15–65)	0.740	39 (15–56)	0.230	47 (36–66)	0.290	49 (29–69)	0.960	60 (51–69)	0.730	49 (44–54)	0.280
	Grade 2	37 (19–60)		37 (21–67)		48 (25–61)		47 (25–56)		51 (–)^a^		67 (–)^a^		67 (–)^a^	
	Grade 3	45 (22–68)		35 (15–68)		35 (15–45)		17 (–)^a^		‐		‐		‐	
Psychiatric comorbidity	No	35 (15–69)	0.760	32 (15–68)	0.160	35 (15–64)	0.240	44 (17–67)	0.440	41 (17–69)	0.910	48 (23–69)	0.710	49 (26–54)	0.450
	Yes	37 (18–52)		38 (15–67)		40 (17–61)		45 (34–56)		41 (32–51)		53 (39–67)		54 (40–67)	
Implicated drugs	CT	36 (15–69)	0.740	35 (15–68)	0.260	35 (15–64)	0.460	43 (17–66)	0.390	37 (17–69)	0.550	46 (23–69)	0.420	42 (26–54)	0.160
	Biologic	30 (15–60)		28 (15–67)		25 (15–53)		52 (48–56)		47 (43–51)		57 (48–67)		58 (49–67)	
Type of CT	Platin	34 (15–69)	0.930	34 (15–68)	0.232	35 (15–61)	0.600	44 (16–66)	0.813	31 (17–69)	0.289	45 (23–69)	0.551	42 (26–54)	0.150
	Non platin	35 (16–64)		31 (15–67)		33 (15–64)		42 (25–56)		43 (41–51)		48 (46–67)		50 (49–67)	

Abbreviations: BTR, breakthrough reaction; CT; chemotherapeutic; DrHy‐Q, Drug Hypersensitivity Quality of Life Questionnaire; max, maximum; min, minimum.

^a^ There is only one value available.

* Bolded *p* < 0.05 was considered statistically significant.

## 4 DISCUSSION

Our results show for the first time the longitudinal changing pattern of DrHy‐Q after up to seven repetitive drug desensitisations in a large sample of patients with drug hypersensitivity. To the best of our knowledge, this is the first study to evaluate the effect of repetitive desensitisations on DrHy‐Q.

There was a significant improvement in DrHy‐Q after the first three desensitisation compared with baseline and this improvement was negatively affected by the development of BTRs during desensitisations. However, repetitive desensitisations did not cause a progressive increase in QOL, as there was no significant improvement in DrHy‐Q after the second and third desensitisations compared with DrHy‐Q after the first desensitisation. In addition, drug hypersensitivity related QOL did not show a change after the fourth and further desensitisations compared to baseline. However, the number of patients with a need of more than three desensitisations was limited to detect an improvement or deterioration in DrHy‐Q scores after the fourth and further desensitisations. Therefore, we cannot reach a clear conclusion regarding the QOL changing pattern after the third desensitisation.

Generic health questionnaires typically assess the physical, social and emotional dimensions of health and tend to have the advantage of assessing QOL for different types of diseases. Disease‐specific QOL questionnaires tend to measure more specific elements of the disease and are therefore theoretically more sensitive to subtle treatment‐related changes than generic QOL measures.^16^, ^17^ Validation studies suggest that DrHy‐Q has the capacity to measure the subjective experiences of patients with DHR that are difficult to measure with generic instruments.^4^, ^7^ In most patients with DHRs, the symptoms are caused by exposure to the responsible drug and the reaction usually disappears after a period when the medication is discontinued. However, patients' efforts to avoid the responsible medications, anxiety and fear of the recurrence of the allergic reaction and concerns about using other drugs can have negative impacts on health related QOL beyond the DHR. Therefore, we used DrHy‐Q in the present study to assess the effect of repeated drug desensitisation.^11^

We showed a significant improvement in drug hypersensitivity related QOL after a single drug desensitisation in a previous study.^11^ On the other hand, the present study specifically evaluated the effects of repeated drug desensitisations and included patients with a need of more than one desensitisation. As a result, the majority of the study population consisted of patients with active malignancy (99/111, 89.2%) and rheumatological diseases (9/111, 8.1%). The median (min–max) baseline DrHy‐Q score of 39 (16–74) observed in this study was similar to our previous study, which was performed in a similar patient group, including patients mainly hypersensitive to chemotherapeutics and biologicals. Conversely, DrHy‐Q scores in the present study were higher than those reported in other previous studies.^4^, ^7^, ^18^, ^19^, ^20^ This difference may be related to the different demographic and clinical characteristics of the study groups. Lower DrHy‐Q scores were reported in studies performed on patients with DHRs mainly due to antibiotics and non‐steroidal anti‐inflammatory drugs (NSAIDs).^4^, ^21^ Hence, the primary disease severity and life‐threatening risk consisting of the indication of the drug implicated in the DHR seem to impact the level of deterioration in drug hypersensitivity related QOL. It was also reported that the increasing severity of the initial DHR, experiencing two and more DHRs, having multiple implicated drugs and drug classes negatively affect drug hypersensitivity related QOL.^7^, ^11^, ^18^, ^22^, ^23^

In the reliability and validity studies of DrHy‐Q conducted in different populations, contrary to our study, the number of patients with DHR to NSAIDs and antibiotics was predominant.^4^, ^20^ In many centres, aspirin desensitisation was a common and recommended treatment modality in patients with a history of recurrent nasal polypectomy and NSAID‐exacerbated airway disease (NERD).^15^, ^21^ However, in recent years, the increase in targeted biological therapies (omalizumab, mepolizumab, dupilumab, etc.) used on nasal polyps has reduced the frequency of aspirin desensitisation, which has a high risk of gastrointestinal side effects.^24^ Therefore, the number of patients desensitised with aspirin has decreased over the years, while the number of patients desensitised with biologics and chemotherapeutics has increased.^25^, ^26^, ^27^

The responsiveness of DrHy‐Q to interventions such as diagnostic tests and finding safe alternative drugs was evaluated in very few studies.^4^, ^11^, ^23^ These studies reported improvements in DHR related QOL with penicillin allergy delabeling, obtaining a conclusive diagnosis with diagnostic allergy work‐up, and finding safe alternative drugs.^4^, ^23^ Our results indicate that the DrHy‐Q scale scores of patients with DHRs significantly improve compared to baseline after up to three repetitive desensitisations. However, no additive effect or progressive improvement was observed with repetitive desensitisations. At the beginning of treatment, concerns of the patients about not being able to use the indicated drug due to hypersensitivity may have negative impacts on our patients' QOL. Although being able to take the culprit drug with desensitisation improved patients' QOL immediately after desensitisation, the lack of progressive improvement with repetitive desensitisations with the same drug may be due to the patients' knowledge that the induced tolerance state is temporary and the need for desensitisation would continue during the continuation of the treatment.

Post‐desensitisation DrHy‐Q scores may be affected by the severity of the initial DHR, indication of the culprit drug, severity of the primary disease, comorbidities, existence of BTRs during desensitisation and severity of these BTRs. BTRs are DHRs which occur despite the desensitisation procedure. They most often occur during the first course of desensitisation and are usually less severe in subsequent procedures.^9^, ^13^, ^14^ In our previous study, we found that improvement in DrHy‐Q after a single desensitisation was not affected by the implicated drugs, indications of the implicated drugs, severity of the initial DHR, and development of a BTR during desensitisation.^11^ In line with this, post‐desensitisation DrHy‐Q scores did not show any significant differences according to the grades of BTRs, existence of a psychiatric comorbidity, or type of the implicated drug in the current study. However, we showed that BTRs during the initial and repetitive desensitisations negatively impact DrHy‐Q, as post‐desensitisation DrHy‐Q scores were higher in patients with BTRs than in those without BTRs in the first three desensitisation, indicating more impairment in QOL. The discrepancy between the effect of BTRs on post‐desensitisation DrHy‐Q scores may be related to the higher rates of BTRs and patients with malignancy requiring repetitive desensitisations in the current study. As far as we know, there is no other study on the effect of BTRs on DrHy‐Q.

The majority of the patients who underwent desensitisation due to drug hypersensitivity in the current study were women. This seems to be related to the prevalence of DHRs in the general population, which is higher in women than in men. There is a growing body of evidence that female gender is associated with an increased risk of immune‐mediated DHRs.^28^, ^29^ The female predominance in the current study may also be related to the frequent use of platins in gynaecological malignancies and the high reaction rates with recurrent treatment cycles of platin compounds. The predominance of chemotherapeutic and biological drug hypersensitivity in the study group was related to the inclusion of the patients with a need of more than one desensitisation. Drug desensitisation induces a temporary state of tolerance, which can only be maintained by the continuous administration of the drug. Therefore, in cases where there is a treatment‐free interval between treatment cycles, such as chemotherapy or biologicals, desensitisation should be repeated before each administration. In addition, the increasing incidence of cancer, as well as the increasing number of chemotherapeutics used in many types of cancer, and the expanding development and use of biologicals have increased the incidence of DHR with both chemotherapeutic and biological drugs. In numerous previous studies, RDD, particularly with chemotherapy drugs, has emerged as a prominent area of interest. RDDs with chemotherapeutic and biological drugs are the most common and promising protocols among desensitisation treatments.^30^, ^31^, ^32^, ^33^, ^34^, ^35^

In conclusion, this study demonstrates for the first time the longitudinal changing pattern of drug hypersensitivity related QOL in repeated drug desensitisations in a large sample. Drug hypersensitivity related QOL evaluated via DrHy‐Q significantly improved during the first three desensitisations compared to baseline, but not after subsequent drug desensitisations, as compared to baseline. The improvement was negatively affected by the development of the BTRs during desensitisation procedures.

## AUTHOR CONTRIBUTIONS

**Begum Gorgulu Akin**: Methodology; investigation; conceptualization; writing—original draft; funding acquisition; writing—review & editing; data curation; validation; visualization; formal analysis; software. **Secil Kepil Ozdemir**: Conceptualization; writing—original draft; funding acquisition; investigation; methodology; writing—review & editing; validation; project administration; formal analysis; supervision; resources; software. **Beyza Doganay Erdogan**: Methodology; formal analysis; project administration; writing—original draft; funding acquisition; investigation; validation; writing—review & editing; data curation. **Asli Gelincik**: Investigation; conceptualization; writing—original draft; funding acquisition; methodology; writing—review & editing; formal analysis. **Ozlem Goksel**: Writing—original draft; writing—review & editing; methodology; validation; investigation; conceptualization. **Adile Berna Dursun**: Conceptualization; investigation; writing—original draft; methodology; writing—review & editing. **Sacide Rana Isık**: Conceptualization; investigation; writing—original draft; methodology; writing—review & editing; formal analysis. **Semra Demir**: Conceptualization; funding acquisition; writing—original draft; methodology; writing—review & editing. **Hatice Serpil Akten**: Conceptualization; investigation; writing—original draft; writing—review & editing; formal analysis; methodology. **Sevim Bavbek**: Methodology; conceptualization; investigation; funding acquisition; writing—original draft; validation; visualization; writing—review & editing; formal analysis; data curation; supervision; resources; project administration; software.

## CONFLICT OF INTEREST STATEMENT

The authors declare no conflicts of interest.

## Supporting information

Table S1

## Data Availability

The authors declare that they have followed the protocols of their work centre on the publication of patient data in the study. All data generated or analysed during this study are included in this article. The data that support the findings of this study are available on request from the corresponding author. The data are not publicly available due to privacy or ethical restrictions.
