# Supplementary material for: The impact of repeated drug desensitisation on quality of life in drug hypersensitivity
Source: Clin Transl Allergy. 2025 Feb 17;15(2):e70029. doi: 10.1002/clt2.70029 (PMC11832298; doi:10.1002/clt2.70029)
Supplement: Supplementary file 1 — Table S1 [file CLT2-15-e70029-s001.docx]

**Supplementary Table S1. Turkish version of the Drug Hypersensitivity Quality of Life Questionnaire**

|  | **Hiç** | **Az** | **Oldukça** | **Çok** | **Çok fazla** |
| --- | --- | --- | --- | --- | --- |
| İlaç kullanamadığım için her hastalık beni diğer insanlardan daha fazla sınırlıyor. | 1 | 2 | 3 | 4 | 5 |
| Acil bir durum söz konusu olduğunda, alerjim olan bir ilacın bana verilmesinden korkuyorum | 1 | 2 | 3 | 4 | 5 |
| İlaçlara karşı olan bu problemim yüzünden kendimi korkmuş hissediyorum | 1 | 2 | 3 | 4 | 5 |
| İlaçlara karşı tepki gösterme problemim hayatımı biçimlendiriyor. | 1 | 2 | 3 | 4 | 5 |
| Diğer uzman doktorlar tarafından yazılan ilaçları kullanmadan önce alerji uzmanının görüşünü almak istiyorum | 1 | 2 | 3 | 4 | 5 |
| Her küçük bir rahatsızlık bile benim için bir problem haline geliyor. | 1 | 2 | 3 | 4 | 5 |
| İlaçları rahat bir şekilde kullanamamam bana kendimi diğerlerinden farklı hissettiriyor | 1 | 2 | 3 | 4 | 5 |
| İlaçlara karşı tepki gösterdiğim için kendimi endişeli hissediyorum. | 1 | 2 | 3 | 4 | 5 |
| Her hastalık için rahatlıkla kullanabileceğim bir ilacın var olduğundan kesin olarak emin olmak isterdim. | 1 | 2 | 3 | 4 | 5 |
| Acı (AĞRI) ile başa çıkamamaktan korkuyorum. | 1 | 2 | 3 | 4 | 5 |
| İlaçlara karşı alerjik reaksiyon gösterme sorunum yüzünden kendimi kederli hissediyorum | 1 | 2 | 3 | 4 | 5 |
| İlaçları rahat bir şekilde kullanamamam bana kendimi diğerlerinden farklı hissettiriyor. | 1 | 2 | 3 | 4 | 5 |
| Bu problemim yüzünden eğlenceden (spor, tatil, yolculuk, vs) vazgeçiyorum | 1 | 2 | 3 | 4 | 5 |
| İlaçlara karşı tepki gösterdiğim için moralim bozuluyor | 1 | 2 | 3 | 4 | 5 |
| İlaç alma zorunluluğu fikri bende sıkıntı duygusu yaratıyor. | 1 | 2 | 3 | 4 | 5 |
